# Percutaneous kyphoplasty for the treatment of diffuse idiopathic skeletal hyperostosis with vertebral fractures: A case report and treatment review

**DOI:** 10.3389/fsurg.2022.922139

**Published:** 2022-07-15

**Authors:** Wenhao Wang, Yixue Huang, Linlin Zhang, Huilin Yang

**Affiliations:** Department of Orthopaedic Surgery, The First Afﬁliated Hospital of Soochow University, Suzhou, China

**Keywords:** diffuse idiopathic skeletal hyperostosis, percutaneous kyphoplasty, vertebral fracture, osteoporosis, treatment

## Abstract

Diffuse idiopathic skeletal hyperostosis (DISH) is a systemic metabolic condition characterized by new bone formation mainly at the anterolateral spine. Surgery such as screw fixation is commonly used for DISH patients who also suffer from vertebral fractures. In this case report, we share a DISH case with lumbar vertebral fracture and osteoporosis who underwent percutaneous kyphoplasty plus braces and medication. Percutaneous kyphoplasty, considered as minimally invasive surgery, may be another treatment option with the advantages of less trauma and faster recovery. The clinical information and radiological findings are described and treatments for DISH with vertebral fractures are then briefly reviewed.

## Introduction

Diffuse idiopathic skeletal hyperostosis (DISH), known as Forestier-Rotes-Querol disease, is a bone-forming condition characterized by the presence of massive bone osteophytes at the spine and joints. The disease mainly affects thoracic vertebrae, but can also affect the cervical and lumbar spine. The incidence rate increases with age and weight gain. DISH may be caused by mechanical factors, but more data shows that metabolism abnormalities are the predisposing factors (1). The symptoms of DISH are mild in the early stage. However, DISH sometimes presents with pain, stiffness, or even dysphagia. A notable feature of DISH is that the radiological findings are severer than clinical symptoms and the diagnosis of DISH is radiographic. Patients with mild symptoms are encouraged to strengthen their muscle strength through appropriate activities. Medical treatments such as non-steroidal anti-inflammatory drugs are also recommended. Surgery may be considered for DISH patients with symptoms of nerve compression or vertebral fractures. Screw internal fixation is widely accepted. However, for elder patients with osteoporosis and cardiopulmonary dysfunction, open surgery is not always the best. Percutaneous kyphoplasty (PKP), regarded as minimally invasive surgery, may be another treatment choice for DISH with vertebral fractures. Here, we reported a DISH case with lumbar fracture and osteoporosis who underwent PKP and received supportive treatments with braces and drugs.

## Case description

**Figure 1:**
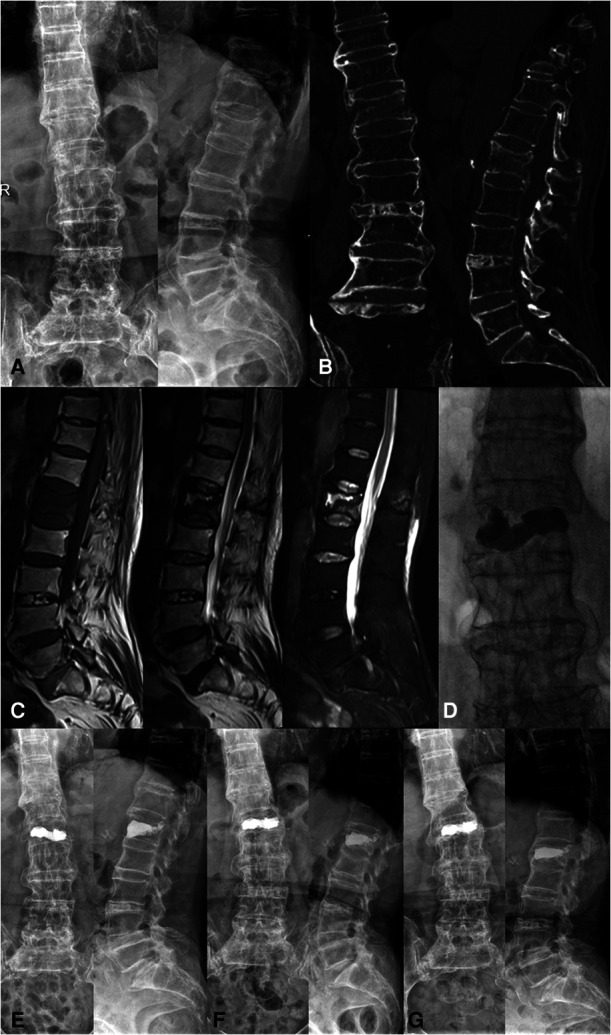
(**A**) X-rays before surgery. (**B**) CT before surgery. Wave-shaped ossification was observed at the anterolateral side of the spine. The L1 fracture was also shown. (**C**) MRI including T1, T2, and STIR before PKP. (**D**) X-rays taken by C-arm fluoroscopy during the operation. Bone cement was injected into the L1 vertebra. (**E)** X-rays at 1 day after surgery. (**F**) X-rays at 2 months after surgery. (**G**) X-rays at 6 months after surgery.

In July 2020, a 75-year-old female came to our hospital and complained about severe low back pain. She fell with her buttocks on the ground 1 month ago. During the month, the low back pain aggravated when she changed positions. Bed rest and oral anti-inflammatory analgesics could slightly relieve the pain. She had Type 2 diabetes for more than 10 years. Physical examination revealed thoracolumbar scoliosis deformity and low back percussion pain. The sensation, muscle strength and muscle tone of both lower extremities were normal. Visual analogue scale (VAS) score was about 6 points. Electrocardiogram suggested atrial fibrillation, and echocardiography showed tricuspid regurgitation and pulmonary hypertension. The results of bone density and biochemical bone markers indicated the diagnosis of osteoporosis. X-rays and computed tomography (CT) showed that wave-shaped ossification was observed at the anterolateral side of at least 4 consecutive segments and osteophytes were formed at the junction of vertebrae and intervertebral disc. Additionally, the L1 vertebra was fractured and the height of the intervertebral space was not changed. Erosion and fusion of the sacroiliac joint were not found (Figures 1A,B). The magnetic resonance imaging (MRI) confirmed the presence of L1 vertebral fracture and the swelling of subcutaneous soft tissue (Figure 1C). Based on all the results above, we made diagnoses of DISH with L1 vertebral fracture and osteoporosis. Considering the patient from all perspectives, we eventually decided to perform PKP with supportive treatments such as braces and medication.[Fig F2]

**Figure 2 F2:**
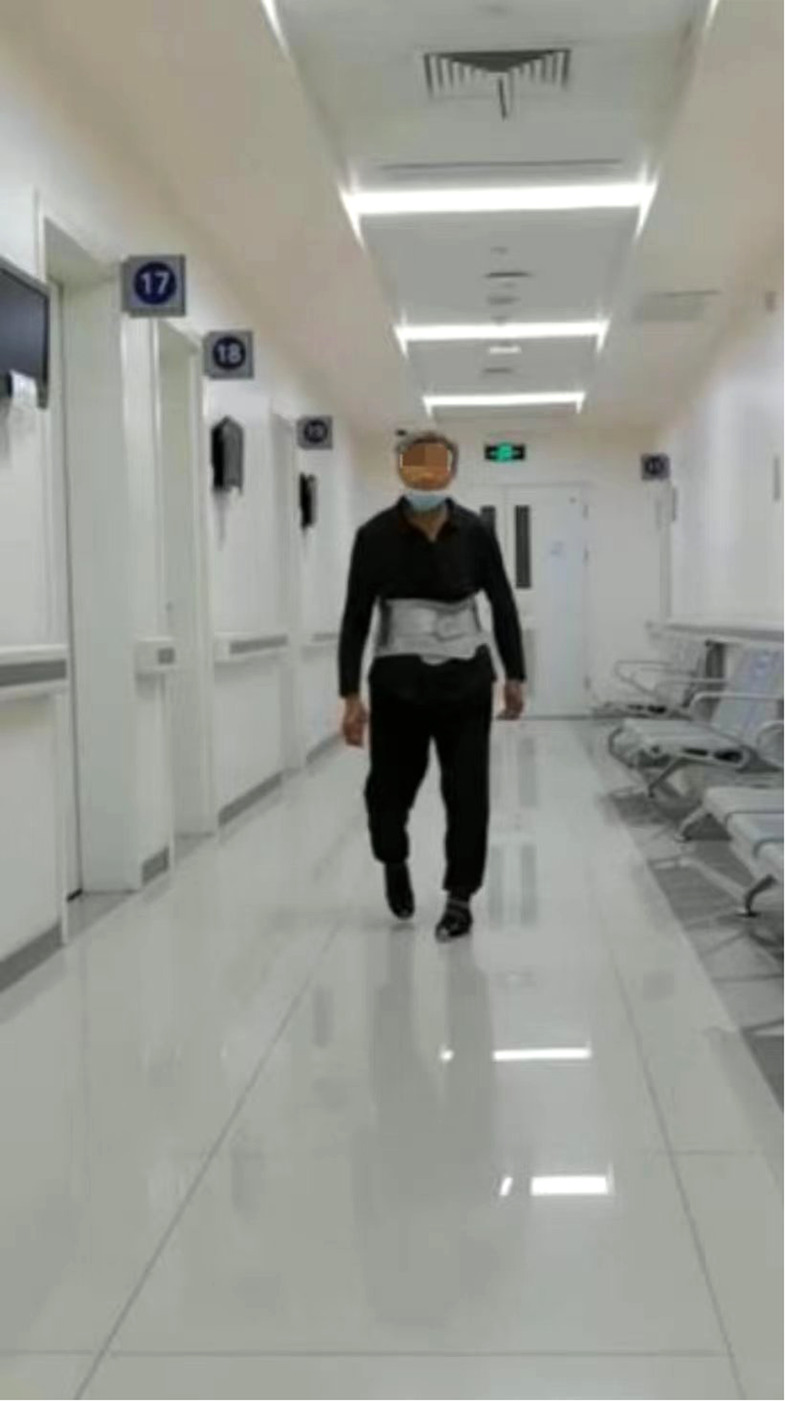
The patient was able to walk with a waist brace after PKP without being bedridden for long periods.

The fractured vertebra guided preoperative preparation. Through preoperative CT 3D reconstruction of the fractured vertebra, we set the direction and location of the bone cement working channel. The cement injection site was located in the cancellous bone, which avoided direct penetration into the fracture fissure. PKP was carried out under general anesthesia and the bilateral approach was adopted through L1 bilateral pedicles. During the surgery, bone cement was perfused in stages because of the temperature difference between the inside and outside of the body. The gradual diffusion of cement could prevent bone cement leakage. The anterior fissure of the vertebral body was first blocked to reduce the cement leakage rate. The posterior edge of the vertebra was observed by lateral fluoroscopy to prevent leakage in the spinal canal. Surgeons could effectively prevent bone cement leakage or displacement by strictly following the surgical procedure. A total of 6 ml bone cement was injected into the fractured vertebra. X-rays were taken by C-arm fluoroscopy during the surgery (Figure 1D). Postoperative VAS score declined to 1 point. After surgery, the patient took calcium carbonate D3 tablets and calcitriol soft capsules orally twice a day. Denosumab was injected subcutaneously every 6 months. X-rays were taken at 1 day, 2 months, and 6 months after surgery, respectively (Figures 1E–G). Bone formation around the fracture was observed in the X-ray at 6 months after surgery especially at the anterior edge of the fractured vertebra. The patient was able to walk with a waist brace after PKP without being bedridden for long periods (Figures 2).

## Discussion

DISH is a skeletal disease characterized by ossification and calcification of ligaments and enthuses, especially at the anterolateral spine and peripheral joints. The prevalence of DISH in the adult population ranges from 2.9% to 42%. The wide range of the prevalence may be due to different characteristics of the study objectives such as age, race, gender, regions, history, and diagnostic criteria. For example, the prevalence of DISH was 3.9% in Africans and 44% in Pacific Islanders. DISH is also associated with risk factors such as obesity, hypertension, atherosclerosis, and diabetes. However, the etiology of DISH remains unclear (1). The typical manifestations of DISH are back pain and spinal stiffness which increase the risk of vertebral fractures. Resnick proposed the diagnostic criteria for DISH in 1975 which has benefited clinical practice (2).

We reviewed the treatments and results on DISH with vertebral fractures in Table 1. Taking drugs and wearing braces are often used as conservative treatments for patients who cannot tolerate surgery (8, 10, 13, 14). Since DISH patients often have age-related complications such as osteoporosis and cardiopulmonary dysfunction, drugs and braces are not always effective and cannot stop the progression of the disease (3, 5, 17). It was also reported that patients wearing braces could suffer from pain and pseudarthrosis, which ultimately resulted in delayed mobility and an increased rate of complications (18). Therefore, conservative treatment alone may not be a recommended treatment option.

**Table 1:** Treatment review on DISH with vertebral fractures.

First author	Year	Treatments	Results and period
Caron T (3)	2010	Anterior or posterior fixation	Effective (mean 6.5 months)
Yeoh D (4)	2014	Percutaneous fixation	Pain-free (mean 22 months)
Westerveld LA (5)	2014	Posterior fixation	Unclear (mean 11.8 months)
Lindtner RA (6)	2017	Percutaneous fixation	Pain relief (1 year)
Okada E (7)	2019	Percutaneous fixation	Effective (1 year)
Murakami Y (8)	2019	Hard corset and posterior fusion	Some benefits (3 months)
Vazan M (9)	2019	Posterior and anterior fixation	Some benefits (mean 2 years)
Chung WH (10)	2020	Posterior fixation and external brace	Effective (6 months)
Kanematsu R (11)	2020	Posterior fixation	Pain relief (6 months)
Ikuma H (12)	2021	Transdiscal screw fixation	Effective (at least 13 months)
Kuroki H (13)	2021	Trunk cast and teriparatide	Pain relief (6 months)
Park HY (14)	2021	Teriparatide	Effective (1 year)
Buxbaum RE (15)	2021	Percutaneous fixation	Effective (at least 1 year)
Uemoto M (16)	2022	Posterior fixation	Unclear (1 month)

Compared with conservative treatment, surgery is more common for DISH patients with vertebral fractures. Spinal fusion is used for DISH patients with unstable three-column fractures and neurological deficits (4, 16). Screw fixation is widely accepted and spinal canal decompression is considered based on the presence of neurological symptoms. Posterior decompression by laminectomy is encouraged if the spinal canal is compressed more than 30% due to fractured fragments or epidural hematoma (9). However, recent studies have reported more screw withdrawal and loosening. This could be prevented by long-segment surgery, which may require the fixation of three vertebrae above and three vertebrae below the fracture segment (6, 11). It is also suggested that the effect of screw fixation can be enhanced by strengthening the screw tunnel with bone cement (15). Furthermore, according to the anatomy of the thoracic and lumbar vertebrae, screws can be used for fixation through the endplate to achieve better stability (12). In recent years, many scholars have focused on minimally invasive surgery such as percutaneous pedicle screw fixation. Unfortunately, DISH patients with vertebral fractures who have undergone screw fixation have more perioperative complications than those tortured by spinal trauma. Buxbaum et al. reported that all patients in their study had at least one postoperative complication (15). Okada et al. reported three patients who died of hypovolemic shock, respiratory failure, and pneumonia within one year after the surgery, respectively (7). For DISH patients with vertebral fractures in poor condition, another option of minimally invasive surgery is badly needed.

PKP, considered as minimally invasive spine surgery, has become popular for the treatment of osteoporotic vertebral compression fractures. Kim et al. reported a case with ankylosing spondylitis and vertebral fractures who underwent PKP and achieved significant postoperative pain relief (19). Another case reported PKP used for a DISH patient with T12 fracture after L2-S1 long-segment fixation (20). Although there are few reports of PKP for DISH patients with vertebral fractures, inspiration and experience can be taken from the cases above. PKP has the advantages of less trauma, short operation time, quick pain relief, and short hospitalization period. In our case, the DISH patient was an old female with cardiovascular diseases which significantly increased the risk of perioperative thrombosis. She also had a long history of Type 2 diabetes which could lead to an increased risk of perioperative infection. The patient also suffered from tricuspid regurgitation and pulmonary hypertension which increased the risk of anaesthesia, especially general anaesthesia. Since the patient was in poor condition, shorter operation time and less trauma were required for the sake of safety. Considering the repeated movement of the fractured fragments could result in osteolysis, instability of the spine, fibrous tissue formation, and neurological deficits, PKP should be performed as soon as possible to achieve better pain relief and early fixation. Additionally, bone cement could fill the fractured vertebra, establish the stability of the anterior and middle columns, minimize damage to the posterior column, and prevent fractures in adjacent segments. Wearing braces ensured early movement within 24 h after surgery thus reducing perioperative complications and mortality. More importantly, the patient also suffered from osteoporosis. Therefore, anti-osteoporosis therapy was adopted to inhibit bone loss. The patient achieved good clinical and radiological outcomes in her 6-month follow-up. Bone formation around the fracture was observed in the X-ray at 6 months after surgery especially at the anterior edge of the vertebra, which showed bone healing and enhanced stability. The patient did not complain pain or difficulty in movement, which showed the treatment alleviated patient's clinical symptoms. PKP plus braces and anti-osteoporosis therapy undoubtedly benefited the DISH patient and greatly improved her quality of life. Although the case treated with PKP is a success, we acknowledge possible complications such as bone cement leakage and pulmonary embolism. Unlike PKP on OVCFs, PKP is not widely used for DISH patients with vertebral fractures and surgeons prefer screw fixation. In addition, vertebral fractures in DISH patients are often accompanied by fissure formation, which is more likely to cause bone cement leakage. This puts forward higher requirements for surgeons' operations. When surgeons treat patients with cement displacement after surgery, percutaneous kyphoplasty can be performed again if the cement is loose as a whole and the surrounding cancellous bone is not firmly riveted. If the cement inside is loose and fragmented, long-segment screw fixation can be adopted. Additionally, waist braces and anti-osteoporosis medication play an important role in the treatment of cement leakage or displacement. This case report provides another treatment option for DISH with vertebral fractures, which is worth sharing. Long-term clinical studies are needed to further evaluate its outcomes and prove its efficacy.

## Conclusions

DISH is a systemic skeletal disease and the underlying mechanism remains unclear. Conservative treatment alone may not be a suitable treatment choice. Surgery is recommended for DISH patients with vertebral fractures, and screw fixation is widely accepted. PKP plus supportive treatments may be another treatment option with the advantages of less trauma, quick pain relief, and short hospital stay. However, the effect of PKP on DISH patients with vertebral fractures still requires further studies with a large sample size and long follow-up.

## Data Availability

The original contributions presented in the study are included in the article/Suplementary Material, further inquiries can be directed to the corresponding author/s.
